# Patient characteristics, pain treatment patterns, and incidence of total joint replacement in a US population with osteoarthritis

**DOI:** 10.1186/s12891-022-05823-7

**Published:** 2022-09-23

**Authors:** Mayura Shinde, Carla Rodriguez-Watson, Tancy C. Zhang, David S. Carrell, Aaron B. Mendelsohn, Young Hee Nam, Amanda Carruth, Kenneth R. Petronis, Cheryl N. McMahill-Walraven, Aziza Jamal-Allial, Vinit Nair, Pamala A. Pawloski, Anne Hickman, Mark T. Brown, Jennie Francis, Ken Hornbuckle, Jeffrey S. Brown, Jingping Mo

**Affiliations:** 1 Harvard Pilgrim Health Care Institute and Harvard Medical School, Boston, MA USA; 2 Reagan-Udall Foundation for the Food and Drug Administration, Washington, DC USA; 3 Kaiser Permanente Washington Health Research Institute, Seattle, WA USA; 4 Pfizer Inc, New York, NY USA; 5 CVS Health Clinical Trial Services (Formerly Known As Healthagen), Blue Bell, PA USA; 6 HealthCore, Inc, Watertown, MA USA; 7 Humana Healthcare Research Inc, Louisville, KY USA; 8 HealthPartners, Bloomington, MN USA; 9 Pfizer Inc, Groton, CT USA; 10 Eli Lilly and Company, Indianapolis, IN USA

**Keywords:** Administrative claims data, Osteoarthritis, Pain medications, Total joint replacement, Treatment patterns, US FDA Sentinel Distributed Database

## Abstract

**Background:**

Currently available medications for chronic osteoarthritis pain are only moderately effective, and their use is limited in many patients because of serious adverse effects and contraindications. The primary surgical option for osteoarthritis is total joint replacement (TJR). The objectives of this study were to describe the treatment history of patients with osteoarthritis receiving prescription pain medications and/or intra-articular corticosteroid injections, and to estimate the incidence of TJR in these patients.

**Methods:**

This retrospective, multicenter, cohort study utilized health plan administrative claims data (January 1, 2013, through December 31, 2019) of adult patients with osteoarthritis in the Innovation in Medical Evidence Development and Surveillance Distributed Database, a subset of the US FDA Sentinel Distributed Database. Patients were analyzed in two cohorts: those with prevalent use of “any pain medication” (prescription non-steroidal anti-inflammatory drugs [NSAIDs], opioids, and/or intra-articular corticosteroid injections) using only the first qualifying dispensing (index date); and those with prevalent use of “each specific pain medication class” with all qualifying treatment episodes identified.

**Results:**

Among 1 992 670 prevalent users of “any pain medication”, pain medications prescribed on the index date were NSAIDs (596 624 [29.9%] patients), opioids (1 161 806 [58.3%]), and intra-articular corticosteroids (323 459 [16.2%]). Further, 92 026 patients received multiple pain medications on the index date, including 71 632 (3.6%) receiving both NSAIDs and opioids. Altogether, 20.6% of patients used an NSAID at any time following an opioid index dispensing and 17.2% used an opioid following an NSAID index dispensing. The TJR incidence rates per 100 person-years (95% confidence interval [CI]) were 3.21 (95% CI: 3.20–3.23) in the “any pain medication” user cohort, and among those receiving “each specific pain medication class” were NSAIDs, 4.63 (95% CI: 4.58–4.67); opioids, 7.45 (95% CI: 7.40–7.49); and intra-articular corticosteroids, 8.05 (95% CI: 7.97–8.13).

**Conclusions:**

In patients treated with prescription medications for osteoarthritis pain, opioids were more commonly prescribed at index than NSAIDs and intra-articular corticosteroid injections. Of the pain medication classes examined, the incidence of TJR was highest in patients receiving intra-articular corticosteroids and lowest in patients receiving NSAIDs.

**Supplementary Information:**

The online version contains supplementary material available at 10.1186/s12891-022-05823-7.

## Background

Recent estimates suggest that about 528 million people worldwide have osteoarthritis (OA) of the hip, knee, hand, shoulder, foot, or other sites [1]. Chronic pain associated with OA has negative effects on health-related quality of life, social function, and work performance [2, 3], and is a major cause of disability [4]. Treatment of chronic OA pain includes non-pharmacologic, pharmacologic, and surgical options [5]. Currently available medications, such as non-steroidal anti-inflammatory drugs (NSAIDs) or opioids, are only moderately effective for treatment of pain in patients with OA, and their use is limited in many patients because of serious adverse effects and contraindications [6–8].

The 2019 guidelines of the American College of Rheumatology/Arthritis Foundation recommend NSAIDs as first-line oral pharmacotherapy for OA pain, at the lowest doses and shortest durations possible because of the risk of adverse cardiovascular, gastrointestinal, and renal side effects. The guidelines only conditionally recommend tramadol versus other opioids, noting the modest benefits and high risk of adverse effects with long-term opioid use [9].

Despite use of available pharmacologic treatments, patients may continue to experience burdensome pain [10]. The primary surgical option for OA is total joint replacement (TJR), and over 95% of TJR procedures are for OA [11]. Among the total US population in 2010, the prevalence of total hip arthroplasty was 0.83% and total knee arthroplasty was 1.52% [12]. The frequency of TJR increases with increasing age; among those ≥ 50 years of age in 2010, the prevalence of total hip arthroplasty was 2.34% and total knee arthroplasty was 4.55% [12].

There is a need to better understand the characteristics, pain treatment patterns, and occurrence of TJR among patients with OA. The objectives of this study were to describe the demographics and clinical characteristics, as well as the pain treatment history, of patients receiving prescription pain medications and/or intra-articular corticosteroid injections for OA, and to estimate the incidence of TJR in these patients.

## Methods

### Study design

This was a population-based, retrospective, multicenter, cohort study of adult patients in the United States with OA receiving prescription pain medications and/or intra-articular corticosteroid injections. The study was conducted using existing health plan administrative claims data from the Innovation in Medical Evidence Development and Surveillance Distributed Database (IMEDS-DD) [13], a subset of the US FDA Sentinel Distributed Database, which is a national electronic system for active surveillance of the safety of medical products in the United States established under the Sentinel Initiative [14–16]. The IMEDS-DD is largely representative of the US commercially insured population and, at present, has claims data available for research for over 95 million health plan members who have medical and pharmacy insurance coverage. The IMEDS-DD leverages the curated data from the Sentinel Common Data Model, along with the data standardization and analytical tools of the Sentinel System for conducting distributed analyses across its network partners [17]. The IMEDS-DD for this study includes six network partners among US national and regional health insurers: Harvard Pilgrim Health Care Institute; HealthCore (Anthem); Humana; Kaiser Permanente Washington Health Research Institute; CVS Health Clinical Trial Services (CVS Health CTS; Aetna); and HealthPartners Institute.

Study cohort eligibility was defined as a diagnosis of OA of the knee, hip, shoulder, or unspecified location on or before the first qualifying dispensing (index date) of a pain medication of interest; age ≥ 18 years on the index date; and continuous health plan enrollment with medical and drug coverage in the 365 days immediately before the index date of pain medication exposure, with allowed enrollment gaps of up to 45 days. To describe patient characteristics and pain treatment history for this study, we defined two pain medication cohorts: patients with prevalent use of “any pain medication” (prescription NSAIDs, opioids, or receiving intra-articular corticosteroids) from January 1, 2013, through December 31, 2019, using only the first qualifying dispensing (index date); and patients with prevalent use of “each specific pain medication class” between January 1, 2013, and December 31, 2019, with all qualifying treatment episodes identified.

### Exposure and outcomes of interest

The pain medications of interest were those commonly prescribed to patients with OA and/or recommended in the 2019 guidelines of the American College of Rheumatology/Arthritis Foundation [9]; these included NSAIDs, opioids, and intra-articular corticosteroids. Pain medication exposures were identified using National Drug Codes and Healthcare Common Procedure Coding System codes recorded in outpatient pharmacy dispensing and medication administration procedures claims data in IMEDS-DD. Pain medication treatment history was evaluated, defined as evidence of prescription pain medication use prior to the index date that did not meet the criteria for index pain medication exposure.

Exposure duration was estimated based on the number of days supplied per dispensing in the pharmacy data. For NSAIDs and opioids, pain medication exposure episodes were created by bridging consecutive pain medication exposure episodes < 30 days apart and adding a 30-day extension to the end of the treatment episode (i.e., based on the assumption that the majority of prescriptions are for 30 days of use) for specific pain medication class subgroups. For intra-articular steroid exposures, a 90-day exposure duration was assigned and consecutive administrations within 30 days were bridged. Sensitivity analyses with 90- and 183-day extensions to the end of the treatment episode were also conducted.

To estimate incidence of TJR in the “any pain medication” cohort, patients were followed irrespective of exposure duration from index dispensing date and until the end of the study period (up to 2555 days [7 years]) unless censored by the first occurrence of any of the following: disenrollment, death, end of the available data (varied across participating health plans), or TJR event.

We also estimated the incidence of TJR during the treatment duration for the “each specific pain medication class” cohort. The follow-up period began on the index date and continued until the first occurrence of any of the following: disenrollment, death, end of the available data, end of the query period, TJR event, discontinuation of treatment, or initiation of a pain medication from a different drug class.

TJR events were identified by International Classification of Diseases, Ninth/Tenth Revision, Clinical Modification (ICD-9-CM/ICD-10-CM) diagnosis codes and/or Current Procedural Terminology codes appearing in claims of any encounter setting, including inpatient, institutional stay, and ambulatory claims. Assessment of TJR did not differentiate knee versus hip TJR events among pain medication users in this analysis. Distributed analyses were performed using the publicly available Sentinel analytic toolkit (Cohort Identification and Descriptive Analysis [CIDA v9.6.0]) [18].

## Results

### Demographics and baseline clinical characteristics

A total of 1 992 670 prevalent users of “any pain medication” were identified between January 1, 2013, and December 31, 2019. Demographics and baseline clinical characteristics for the “any pain medication” cohort are shown in Table 1. The mean (standard deviation) age was 68.4 (12.3) years, and 59.0% were female. Comorbidities identified during the year prior to index date included cardiovascular disease in 70.7%, diabetes in 27.5%, obesity in 19.0%, gastrointestinal hemorrhage in 17.8%, chronic kidney disease in 17.3%, depression in 16.4%, and osteoporosis in 10.1% of patients.

**Table 1:** Demographics and baseline characteristics of patients in the “any pain medication” cohort

Characteristic	*N* = 1 992 670
Age, mean (SD), years	68.4 (12.3)
Age group, *n* (%)	
18–44 years	87 110 (4.4)
45–64 years	648 388 (32.5)
65–74 years	627 246 (31.5)
≥ 75 years	629 926 (31.6)
Sex, *n* (%)	
Female	1 176 078 (59.0)
Male	816 557 (41.0)
Missing	35 (0.0)
Comorbidities ^a^ during year prior to index, *n* (%)	
Cardiovascular disease	1 407 848 (70.7)
Diabetes	547 181 (27.5)
Obesity	378 799 (19.0)
Gastrointestinal hemorrhage	354 631 (17.8)
Chronic kidney disease	344 424 (17.3)
Depression	327 448 (16.4)
Osteoporosis	200 734 (10.1)
Gout	104 686 (5.3)

*SD* standard deviation

^a^Recorded in ≥ 5% of patients

### Pain medication treatment history and treatment patterns

Among the “any pain medication” users, history of ≥ 1 dispensing of NSAIDs was noted in 45.5%, opioids in 62.6%, and intra-articular corticosteroids in 25.0% of patients during the entire available history prior to index date (Table 2).

**Table 2:** Prescription pain medication treatment patterns in the “any pain medication” cohort ^a^

Treatment pattern	*n* (%)
Historic use of prescription pain medication prior to index date ^b^	
NSAID	906 909 (45.5)
Opioid	1 248 379 (62.6)
Intra-articular corticosteroid	497 987 (25.0)
Index pain medication ^c^	
NSAID	596 624 (29.9)
Opioid	1 161 806 (58.3)
Intra-articular corticosteroid	323 459 (16.2)
Multiple pain medications at index	
NSAID and opioid	71 632 (3.6)
NSAID and intra-articular corticosteroid	13 278 (0.7)
Opioid and intra-articular corticosteroid	6286 (0.3)
NSAID, opioid, and intra-articular corticosteroid	830 (0.0)
Subsequent pain medication dispensing within 7 days after index date	
NSAID followed by an opioid	22 397 (1.1)
NSAID followed by an intra-articular corticosteroid	3924 (0.2)
NSAID followed by an opioid and an intra-articular corticosteroid	357 (0.0)
Opioid followed by an NSAID	18 944 (1.0)
Opioid followed by an intra-articular corticosteroid	6383 (0.3)
Opioid followed by an NSAID and an intra-articular corticosteroid	456 (0.0)
Intra-articular corticosteroid followed by an NSAID	4505 (0.2)
Intra-articular corticosteroid followed by an opioid	5370 (0.3)
Intra-articular corticosteroid followed by an NSAID and an opioid	469 (0.0)
Subsequent pain medication dispensing at any time after index date	
NSAID followed by an opioid	342 430 (17.2)
NSAID followed by an intra-articular corticosteroid	147 301 (7.4)
NSAID followed by an opioid and an intra-articular corticosteroid	116 274 (5.8)
Opioid followed by an NSAID	409 829 (20.6)
Opioid followed by an intra-articular corticosteroid	259 299 (13.0)
Opioid followed by an NSAID and an intra-articular corticosteroid	147 148 (7.4)
Intra-articular corticosteroid followed by an NSAID	96 477 (4.8)
Intra-articular corticosteroid followed by an opioid	154 120 (7.7)
Intra-articular corticosteroid followed by an NSAID and an opioid	72 840 (3.7)

*NSAID* non-steroidal anti-inflammatory drug

^a^*N* = 1 992 670

^b^Historic use included patients with > 1 dispensing of pain medication use prior to the index date that did not meet the other inclusion criteria for index pain medication exposure, most notably the requirement to have a 365-day baseline history period prior to the index date

^c^Index pain medication represents the first qualifying dispensing of any pain medication including NSAIDs, opioids, and intra-articular corticosteroids during the study period

Examining the pain medications prescribed on the index date in the “any pain medication” cohort, 596 624 (29.9%) patients received NSAIDs, 1 161 806 (58.3%) received opioids, and 323 459 (16.2%) patients received intra-articular corticosteroids (Table 2). Further, on the index date, a total of 92 026 patients received multiple pain medications, including 71 632 (3.6%) patients who received both NSAIDs and opioids. Although < 5% of patients had a subsequent dispensing of a different class of pain medication within 7 days after the index date, 20.6% of patients used an NSAID at any time following an opioid index dispensing and 17.2% used an opioid at any time following an NSAID index dispensing (Table 2).

Among prescription pain medication recipients in the “each specific pain medication class” cohort, index pain medications were NSAIDs in 1 947 237 patients, opioids in 2 726 480 patients, and intra-articular corticosteroids in 1 320 838 patients. The mean age and sex distributions were similar across these groups, as shown in Supplementary Table S1 (see Additional file 1). Opioid users had a higher prevalence of certain chronic health conditions (cardiovascular disease, chronic kidney disease, and diabetes) compared with NSAID users and patients receiving intra-articular corticosteroids.

### Incidence of total joint replacement

In the “any pain medication” cohort, the TJR incidence rate per 100 person-years (95% confidence interval [CI]) was 3.21 (95% CI: 3.20–3.23; Table 3).

**Table 3:** Incidence rates of TJR events per 100 person-years

Cohort	Number of episodes	Episodes with TJR event	Incidence rate ^a^ (95% confidence interval)
“**Any pain medication**”, with up to 7 years of follow-up ^b^	1 992 670	183 093	3.21 (3.20–3.23)
“**Each specific pain medication class**”			
NSAID ^c^	1 947 237	36 369	4.63 (4.58–4.67)
Opioid ^c^	2 726 480	89 748	7.45 (7.40–7.49)
Intra-articular corticosteroid ^c^	1 320 838	37 980	8.05 (7.97–8.13)

*NSAID* non-steroidal anti-inflammatory drug

*TJR* total joint replacement

^a^Incidence rate was calculated as episodes with a TJR event divided by person-years at risk times 100

^b^For prevalent users of any prescription pain medication, a follow-up time of up to 7 years after index date was assigned for evaluation of TJR occurrence. Only the first qualifying dispensing (index) for each health plan member was included; cohort re‐entry was not allowed. Person‐years at risk was censored at occurrence of death, disenrollment, query end date, network partner end date, and/or TJR occurrence

^c^For prevalent users of each specific drug class, all qualifying dispensings were identified. TJR was assessed during the treatment duration with a 30-day extension to the end of the treatment episode for the NSAID and opioid subgroups, and using a pre-specified 90-day treatment duration for the intra-articular corticosteroid subgroup with a 30-day extension. Person‐years at risk was censored at evidence of death, disenrollment, query end date, network partner end date, TJR occurrence, discontinuation of treatment, and/or initiation of another class of pain medication (e.g., treatment episodes for NSAID users were censored at initiation of opioid or intra‐articular corticosteroid use)

Among patients in the “each specific pain medication class” cohort, the TJR incidence rates per 100 person-years (95% CI) were 4.63 (95% CI: 4.58–4.67) for NSAIDs, 7.45 (95% CI: 7.40–7.49) for opioids, and 8.05 (95% CI: 7.97–8.13) for intra-articular corticosteroids, during the treatment duration, with a 30-day extension to the end of the treatment episode for the NSAID and opioid users, and using a 90-day exposure duration and 30-day extension for those receiving intra-articular corticosteroids (Table 3).

## Discussion

In this retrospective study of nearly 2 million US patients treated with prescription medications for OA pain, opioids were more commonly used than NSAIDs and intra-articular corticosteroid injections. We found an overall TJR incidence rate of 3.21 per 100 person-years, and the incidence rate of TJR was highest in patients receiving intra-articular corticosteroids and lowest in patients receiving NSAIDs.

Our analysis found a high prevalence of comorbid conditions, including cardiovascular disease, diabetes, obesity, and osteoporosis, in patients with OA receiving pain medications. Similarly, a 2011 claims database cohort analysis by Gore et al. showed that patients with OA had a significantly higher prevalence of comorbidities and greater use of pain medications compared with a control cohort [19]. Postler et al. conducted a cross-sectional study of German claims data and found comorbidities were common among 595 754 patients with hip or knee OA (e.g., arterial hypertension in 78.7%, diabetes in 29.0%, and depression in 27.9%) [20]. Their study showed that 63.4% of patients with OA were prescribed analgesics and 44.1% were prescribed NSAIDs [20].

Currently, NSAIDs are first-line pharmacologic agents for management of pain associated with OA; this is despite their associated risks [9, 21–23]. Growing recognition of the risks associated with non-tramadol opioids has led to conditional recommendations against their use [9, 21–23]. One recent, large observational study by Nalamachu et al. reported that 710 of 841 (84%) commercially insured patients visiting physicians for treatment of OA had been prescribed a pain medication [5]. Their analysis found that patients with severe OA pain were more likely than those with mild or moderate pain to have been prescribed opioids and multiple medications, and > 80% of treatment switches were because of lack of efficacy [5].

In contrast to our current analysis, which found opioids were the most commonly prescribed treatment at index date in 62.6% of the “any pain medication” cohort, a 2021 retrospective US electronic health records analysis of adults with a new diagnosis of knee OA found that the most frequent first treatment was intra-articular corticosteroids, in 25.5% of patients, and the most frequent second treatment was opioids, in 15.8% [24]. However, our analysis was not limited to patients with newly diagnosed OA, which may explain the treatment difference. Analyses of data from patients with knee OA in the South Korean nationwide claims database showed that 12.2% received opioids (most commonly tramadol) as their first treatment [25] and 82.5% received an NSAID at any point [26]. Notably, a retrospective longitudinal study of US insurance claims data found that among patients with hip or knee OA who received opioids, 34.9% had evidence of opioid regimen failure, as suggested by an increase in regimen intensity, additional non-opioid pain medication, opioid abuse events, or joint surgery [10]. Our current analysis also found that a substantial proportion (20.6%) of patients who received an opioid at index date subsequently received an NSAID.

An analysis by Berger et al. of US health insurance data for patients with OA who had undergone a TJR showed that in the 2 years prior to hip or knee TJR, 55.2% had received prescription NSAIDs, 58.4% had received opioids, and 50.3% had received intra-articular corticosteroid injections [27]. Similarly, an observational cohort study by Jin et al. of Medicare patients who underwent hip or knee TJR showed that 60.2% had received opioids in the 12 months before surgery [28].

Our current analysis of data from patients being treated for OA-related pain between 2013 and 2019 found an overall TJR incidence rate per 100 person-years of 3.21. A 2011 review of trends in knee and hip TJR procedures performed for OA found that TJR rates varied by country, have increased over the preceding 2–3 decades, and were predicted to increase further due to improved longevity and an aging population [29]. The cross-sectional study of German claims data by Postler et al. showed that 5.3% of patients with hip or knee OA underwent a TJR in 2014 [20]. Among Danish postmenopausal women, the incidence rates of knee and hip TJR increased with age up to 80–84 years of age, when the yearly average incidence rates reached 64 per 10 000 population for knee TJR and 115 per 10 000 population for hip TJR [30].

A possible limitation of our findings is the use of claims-based algorithms to define exposures, outcomes, and baseline characteristics, which may have caused some patients to be misclassified. In addition, there was a lack of data available for use of non-prescription pain medication (including non-prescription NSAIDs), complementary and alternative medicines, and homeopathic remedies. Further, chronic pain may be a driver for increased health care utilization, and we would expect to see increased utilization among those with chronic pain [31]. In addition, we described pain medication use among primarily commercially insured patients, and our results may not be comparable to patients with publicly insured health plans [32].

## Conclusions

Among this population of nearly 2 million individuals in the United States receiving prescription medications for OA pain from 2013 until the end of 2019 (prior to the COVID-19 pandemic), opioids were more commonly used than NSAIDs and intra-articular corticosteroid injections. Further, 41% of opioid users and 30% of NSAID users at index date subsequently received a different class of pain medication. Of the specific pain medication classes examined, the incidence rate of TJR was highest in patients receiving intra-articular corticosteroids and lowest in patients receiving NSAIDs.

## Supplementary Information

**Additional file 1: Supplementary Table S1.** Demographics and baseline clinical characteristics for prescription pain medication recipients in the “each specific pain medication class” cohort.

**Additional file 2: Appendix D.** List of International Classification of Diseases, Ninth Revision, Clinical Modification (ICD-9-CM), International Classification of Diseases, Tenth Revision, Clinical Modification (ICD-10-CM), and Current Procedural Terminology, Fourth Edition (CPT-4) Procedure Codes Used to Define the Outcome (Aims 1-3) and Exposure (Aim 4) in this Request.

## Data Availability

The data that support the findings of this study are available from the corresponding author but restrictions apply to the availability of these data, which were used under license for the current study, and so are not publicly available. Data are however available from the authors upon reasonable request and with permission of Reagan-Udall Foundation for the FDA.

## Abbreviations

CI: Confidence interval
ICD-9-CM: International Classification of Diseases, 9^th^ Revision, Clinical Modification
ICD-10-CM: International Classification of Diseases, 10^th^ Revision, Clinical Modification
IMEDS-DD: Innovation in Medical Evidence Development and Surveillance Distributed Database
NSAID: Non-steroidal anti-inflammatory drug
OA: Osteoarthritis
SD: Standard deviation
TJR: Total joint replacement
