# Supplementary material for: Patient characteristics, pain treatment patterns, and incidence of total joint replacement in a US population with osteoarthritis
Source: BMC Musculoskelet Disord. 2022 Sep 23;23:883. doi: 10.1186/s12891-022-05823-7 (PMC9502954; doi:10.1186/s12891-022-05823-7)
Supplement: Supplementary file 1 — Additional file 1: Supplementary Table S1. Demographics and baseline clinical characteristics for prescription pain medication recipients in the “each specific pain medication class” cohort. [file 12891_2022_5823_MOESM1_ESM.docx]

**Supplementary Table S1** Demographics and baseline clinical characteristics for prescription pain medication recipients in the “each specific pain medication class” cohort

| Characteristic | NSAID  (*n* = 1 947 237) | Opioid  (*n* = 2 726 480) | Intra-articular corticosteroid  (*n* = 1 320 838) |
| --- | --- | --- | --- |
| Total episodes | 4 296 389 | 7 793 457 | 2 215 050 |
| Age, years, mean (SD) | 66.1 (11.5) | 69.2 (11.7) | 69.2 (11.3) |
| Age group, *n* (%) | |  |  |
| 18–44 years | 206 869 (4.8) | 260 078 (3.3) | 58 134 (2.6) |
| 45–64 years | 1 668 327 (38.8) | 2 389 662 (30.7) | 692 866 (31.3) |
| 65–74 years | 1 427 084 (33.2) | 2 555 221 (32.8) | 743 663 (33.6) |
| ≥75 years | 994 109 (23.1) | 2 588 496 (33.2) | 720 387 (32.5) |
| Sex, *n* (%) |  |  |  |
| Female | 1 187 855 (61.0) | 1 619 817 (59.4) | 814 089 (61.6) |
| Male | 759 359 (39.0) | 1 106 620 (40.6) | 506 734 (38.4) |
| Missing | 23 (0.0) | 43 (0.0) | 15 (0.0) |
| Comorbidities ^a^ during year prior to index, *n* (%) | |  |  |
| Cardiovascular disease | 3 082 090 (71.7) | 6 145 348 (78.9) | 1 658 126 (74.9) |
| Diabetes | 1 245 825 (29.0) | 2 640 606 (33.9) | 630 125 (28.4) |
| Obesity | 1 273 701 (29.6) | 2 337 140 (30.0) | 647 305 (29.2) |
| Gastrointestinal hemorrhage | 770 622 (17.9) | 1 698 183 (21.8) | 425 784 (19.2) |
| Chronic kidney disease | 740 834 (17.2) | 2 070 455 (26.6) | 457 318 (20.6) |
| Depression | 920 278 (21.4) | 1 882 768 (24.2) | 467 971 (21.1) |
| Osteoporosis | 405 931 (9.4) | 878 392 (11.3) | 237 852 (10.7) |
| Gout | 283 345 (6.6) | 554 888 (7.1) | 134 230 (6.1) |
| Rheumatoid arthritis | 209 390 (4.9) | 416 954 (5.4) | 125 743 (5.7) |

*NSAID* non-steroidal anti-inflammatory drug

*SD* standard deviation

^a^ Recorded in ≥5% of patients in any treatment group
