# Supplementary material for: Patient characteristics, pain treatment patterns, and incidence of total joint replacement in a US population with osteoarthritis
Source: BMC Musculoskelet Disord. 2022 Sep 23;23:883. doi: 10.1186/s12891-022-05823-7 (PMC9502954; doi:10.1186/s12891-022-05823-7)
Supplement: Supplementary file 2 — Additional file 2: Appendix D. List of International Classification of Diseases, Ninth Revision, Clinical Modification (ICD-9-CM), International Classification of Diseases, Tenth Revision, Clinical Modification (ICD-10-CM), and Current Procedural Terminology, Fourth Edition (CPT-4) Procedure Codes Used to Define the Outcome (Aims 1-3) and Exposure (Aim 4) in this Request. [file 12891_2022_5823_MOESM2_ESM.docx]

| **Appendix D. List of International Classification of Diseases, Ninth Revision, Clinical Modification (ICD-9-CM), International Classification of Diseases, Tenth Revision, Clinical Modification (ICD-10-CM), and Current Procedural Terminology, Fourth Edition (CPT-4) Procedure Codes Used to Define the Outcome (Aims 1-3) and Exposure (Aim 4) in this Request** | | | |
| --- | --- | --- | --- |
|  |  |  |  |
| **Code** | **Description** | **Code Type** | **Code Category** |
| **Total Joint Replacement** | | | |
| 23472 | RECONSTRUCT SHOULDER JOINT | CPT-4 | Procedure |
| 27130 | TOTAL HIP ARTHROPLASTY | CPT-4 | Procedure |
| 27132 | TOTAL HIP ARTHROPLASTY | CPT-4 | Procedure |
| 27445 | REVISION OF KNEE JOINT | CPT-4 | Procedure |
| 27447 | TOTAL KNEE ARTHROPLASTY | CPT-4 | Procedure |
| 81.51 | Total hip replacement | ICD-9-CM | Procedure |
| 81.54 | Total knee replacement | ICD-9-CM | Procedure |
| 81.80 | Other total shoulder replacement | ICD-9-CM | Procedure |
| 0SRD0LZ | Replacement of Left Knee Joint with Medial Unicondylar Synthetic Substitute, Open Approach | ICD-10-CM | Procedure |
| 0SRD0L9 | Replacement of Left Knee Joint with Medial Unicondylar Synthetic Substitute, Cemented, Open Approach | ICD-10-CM | Procedure |
| 0RRK0J6 | Replacement of Left Shoulder Joint with Synthetic Substitute, Humeral Surface, Open Approach | ICD-10-CM | Procedure |
| 0SRC0LZ | Replacement of Right Knee Joint with Medial Unicondylar Synthetic Substitute, Open Approach | ICD-10-CM | Procedure |
| 0SRD069 | Replacement of Left Knee Joint with Oxidized Zirconium on Polyethylene Synthetic Substitute, Cemented, Open Approach | ICD-10-CM | Procedure |
| 0SR902A | Replacement of Right Hip Joint with Metal on Polyethylene Synthetic Substitute, Uncemented, Open Approach | ICD-10-CM | Procedure |
| 0RRJ00Z | Replacement of Right Shoulder Joint with Reverse Ball and Socket Synthetic Substitute, Open Approach | ICD-10-CM | Procedure |
| 0RRK0KZ | Replacement of Left Shoulder Joint with Nonautologous Tissue Substitute, Open Approach | ICD-10-CM | Procedure |
| 0RRK00Z | Replacement of Left Shoulder Joint with Reverse Ball and Socket Synthetic Substitute, Open Approach | ICD-10-CM | Procedure |
| 0RRJ0KZ | Replacement of Right Shoulder Joint with Nonautologous Tissue Substitute, Open Approach | ICD-10-CM | Procedure |
| 0SRC0J9 | Replacement of Right Knee Joint with Synthetic Substitute, Cemented, Open Approach | ICD-10-CM | Procedure |
| 0SRC06A | Replacement of Right Knee Joint with Oxidized Zirconium on Polyethylene Synthetic Substitute, Uncemented, Open Approach | ICD-10-CM | Procedure |
| 0SRC06Z | Replacement of Right Knee Joint with Oxidized Zirconium on Polyethylene Synthetic Substitute, Open Approach | ICD-10-CM | Procedure |
| 0SR90KZ | Replacement of Right Hip Joint with Nonautologous Tissue Substitute, Open Approach | ICD-10-CM | Procedure |
| 0SRB01Z | Replacement of Left Hip Joint with Metal Synthetic Substitute, Open Approach | ICD-10-CM | Procedure |
| 0RRJ07Z | Replacement of Right Shoulder Joint with Autologous Tissue Substitute, Open Approach | ICD-10-CM | Procedure |
| 0SR907Z | Replacement of Right Hip Joint with Autologous Tissue Substitute, Open Approach | ICD-10-CM | Procedure |
| 0SRB0J9 | Replacement of Left Hip Joint with Synthetic Substitute, Cemented, Open Approach | ICD-10-CM | Procedure |
| 0SRC07Z | Replacement of Right Knee Joint with Autologous Tissue Substitute, Open Approach | ICD-10-CM | Procedure |
| 0SR903A | Replacement of Right Hip Joint with Ceramic Synthetic Substitute, Uncemented, Open Approach | ICD-10-CM | Procedure |
| 0SRB0JA | Replacement of Left Hip Joint with Synthetic Substitute, Uncemented, Open Approach | ICD-10-CM | Procedure |
| 0SRC0JA | Replacement of Right Knee Joint with Synthetic Substitute, Uncemented, Open Approach | ICD-10-CM | Procedure |
| 0SR901Z | Replacement of Right Hip Joint with Metal Synthetic Substitute, Open Approach | ICD-10-CM | Procedure |
| 0SRD06Z | Replacement of Left Knee Joint with Oxidized Zirconium on Polyethylene Synthetic Substitute, Open Approach | ICD-10-CM | Procedure |
| 0SRD07Z | Replacement of Left Knee Joint with Autologous Tissue Substitute, Open Approach | ICD-10-CM | Procedure |
| 0SR90JA | Replacement of Right Hip Joint with Synthetic Substitute, Uncemented, Open Approach | ICD-10-CM | Procedure |
| 0RRJ0J7 | Replacement of Right Shoulder Joint with Synthetic Substitute, Glenoid Surface, Open Approach | ICD-10-CM | Procedure |
| 0SRC069 | Replacement of Right Knee Joint with Oxidized Zirconium on Polyethylene Synthetic Substitute, Cemented, Open Approach | ICD-10-CM | Procedure |
| 0SRB07Z | Replacement of Left Hip Joint with Autologous Tissue Substitute, Open Approach | ICD-10-CM | Procedure |
| 0RRJ0J6 | Replacement of Right Shoulder Joint with Synthetic Substitute, Humeral Surface, Open Approach | ICD-10-CM | Procedure |
| 0SRB06A | Replacement of Left Hip Joint with Oxidized Zirconium on Polyethylene Synthetic Substitute, Uncemented, Open Approach | ICD-10-CM | Procedure |
| 0SR904Z | Replacement of Right Hip Joint with Ceramic on Polyethylene Synthetic Substitute, Open Approach | ICD-10-CM | Procedure |
| 0SR9069 | Replacement of Right Hip Joint with Oxidized Zirconium on Polyethylene Synthetic Substitute, Cemented, Open Approach | ICD-10-CM | Procedure |
| 0SRB02A | Replacement of Left Hip Joint with Metal on Polyethylene Synthetic Substitute, Uncemented, Open Approach | ICD-10-CM | Procedure |
| 0SRB069 | Replacement of Left Hip Joint with Oxidized Zirconium on Polyethylene Synthetic Substitute, Cemented, Open Approach | ICD-10-CM | Procedure |
| 0SRB03Z | Replacement of Left Hip Joint with Ceramic Synthetic Substitute, Open Approach | ICD-10-CM | Procedure |
| 0RRK07Z | Replacement of Left Shoulder Joint with Autologous Tissue Substitute, Open Approach | ICD-10-CM | Procedure |
| 0SR906A | Replacement of Right Hip Joint with Oxidized Zirconium on Polyethylene Synthetic Substitute, Uncemented, Open Approach | ICD-10-CM | Procedure |
| 0SRD0LA | Replacement of Left Knee Joint with Medial Unicondylar Synthetic Substitute, Uncemented, Open Approach | ICD-10-CM | Procedure |
| 0RRK0JZ | Replacement of Left Shoulder Joint with Synthetic Substitute, Open Approach | ICD-10-CM | Procedure |
| 0SRB06Z | Replacement of Left Hip Joint with Oxidized Zirconium on Polyethylene Synthetic Substitute, Open Approach | ICD-10-CM | Procedure |
| 0SRB04A | Replacement of Left Hip Joint with Ceramic on Polyethylene Synthetic Substitute, Uncemented, Open Approach | ICD-10-CM | Procedure |
| 0RRJ0JZ | Replacement of Right Shoulder Joint with Synthetic Substitute, Open Approach | ICD-10-CM | Procedure |
| 0SRB019 | Replacement of Left Hip Joint with Metal Synthetic Substitute, Cemented, Open Approach | ICD-10-CM | Procedure |
| 0SR90JZ | Replacement of Right Hip Joint with Synthetic Substitute, Open Approach | ICD-10-CM | Procedure |
| 0SRD0KZ | Replacement of Left Knee Joint with Nonautologous Tissue Substitute, Open Approach | ICD-10-CM | Procedure |
| 0SRC0KZ | Replacement of Right Knee Joint with Nonautologous Tissue Substitute, Open Approach | ICD-10-CM | Procedure |
| 0SRC0JZ | Replacement of Right Knee Joint with Synthetic Substitute, Open Approach | ICD-10-CM | Procedure |
| 0SRB04Z | Replacement of Left Hip Joint with Ceramic on Polyethylene Synthetic Substitute, Open Approach | ICD-10-CM | Procedure |
| 0SRD0JZ | Replacement of Left Knee Joint with Synthetic Substitute, Open Approach | ICD-10-CM | Procedure |
| 0SRD06A | Replacement of Left Knee Joint with Oxidized Zirconium on Polyethylene Synthetic Substitute, Uncemented, Open Approach | ICD-10-CM | Procedure |
| 0RRK0J7 | Replacement of Left Shoulder Joint with Synthetic Substitute, Glenoid Surface, Open Approach | ICD-10-CM | Procedure |
| 0SR901A | Replacement of Right Hip Joint with Metal Synthetic Substitute, Uncemented, Open Approach | ICD-10-CM | Procedure |
| 0SR904A | Replacement of Right Hip Joint with Ceramic on Polyethylene Synthetic Substitute, Uncemented, Open Approach | ICD-10-CM | Procedure |
| 0SRC0LA | Replacement of Right Knee Joint with Medial Unicondylar Synthetic Substitute, Uncemented, Open Approach | ICD-10-CM | Procedure |
| 0SRB029 | Replacement of Left Hip Joint with Metal on Polyethylene Synthetic Substitute, Cemented, Open Approach | ICD-10-CM | Procedure |
| 0SRB049 | Replacement of Left Hip Joint with Ceramic on Polyethylene Synthetic Substitute, Cemented, Open Approach | ICD-10-CM | Procedure |
| 0SRB03A | Replacement of Left Hip Joint with Ceramic Synthetic Substitute, Uncemented, Open Approach | ICD-10-CM | Procedure |
| 0SR9019 | Replacement of Right Hip Joint with Metal Synthetic Substitute, Cemented, Open Approach | ICD-10-CM | Procedure |
| 0SRC0L9 | Replacement of Right Knee Joint with Medial Unicondylar Synthetic Substitute, Cemented, Open Approach | ICD-10-CM | Procedure |
| 0SRB02Z | Replacement of Left Hip Joint with Metal on Polyethylene Synthetic Substitute, Open Approach | ICD-10-CM | Procedure |
| 0SR9029 | Replacement of Right Hip Joint with Metal on Polyethylene Synthetic Substitute, Cemented, Open Approach | ICD-10-CM | Procedure |
| 0SRB0JZ | Replacement of Left Hip Joint with Synthetic Substitute, Open Approach | ICD-10-CM | Procedure |
| 0SR906Z | Replacement of Right Hip Joint with Oxidized Zirconium on Polyethylene Synthetic Substitute, Open Approach | ICD-10-CM | Procedure |
| 0SRB0KZ | Replacement of Left Hip Joint with Nonautologous Tissue Substitute, Open Approach | ICD-10-CM | Procedure |
| 0SR9039 | Replacement of Right Hip Joint with Ceramic Synthetic Substitute, Cemented, Open Approach | ICD-10-CM | Procedure |
| 0SRB01A | Replacement of Left Hip Joint with Metal Synthetic Substitute, Uncemented, Open Approach | ICD-10-CM | Procedure |
| 0SRD0J9 | Replacement of Left Knee Joint with Synthetic Substitute, Cemented, Open Approach | ICD-10-CM | Procedure |
| 0SR9049 | Replacement of Right Hip Joint with Ceramic on Polyethylene Synthetic Substitute, Cemented, Open Approach | ICD-10-CM | Procedure |
| 0SR903Z | Replacement of Right Hip Joint with Ceramic Synthetic Substitute, Open Approach | ICD-10-CM | Procedure |
| 0SRD0JA | Replacement of Left Knee Joint with Synthetic Substitute, Uncemented, Open Approach | ICD-10-CM | Procedure |
| 0SRB039 | Replacement of Left Hip Joint with Ceramic Synthetic Substitute, Cemented, Open Approach | ICD-10-CM | Procedure |
| 0SR902Z | Replacement of Right Hip Joint with Metal on Polyethylene Synthetic Substitute, Open Approach | ICD-10-CM | Procedure |
| 0SR90J9 | Replacement of Right Hip Joint with Synthetic Substitute, Cemented, Open Approach | ICD-10-CM | Procedure |
